# Brain region-specific genome-wide deoxyribonucleic acid methylation analysis in patients with Alzheimer’s disease

**DOI:** 10.3389/fnmol.2023.971565

**Published:** 2023-04-13

**Authors:** Gang Ren, Shan Song, Sheng-Xiao Zhang, Yan Liu, Yan Lv, Yan-Hong Wang, Rong Zhao, Xin-Yi Li

**Affiliations:** ^1^Department of Neurology, Third Hospital of Shanxi Medical University, Shanxi Bethune Hospital, Shanxi Academy of Medical Sciences, Tongji Shanxi Hospital, Taiyuan, China; ^2^Department of Rheumatology, The Second Hospital of Shanxi Medical University, Taiyuan, China; ^3^Key Laboratory of Cellular Physiology at Shanxi Medical University, Ministry of Education, Taiyuan, China; ^4^Department of Nephrology, Third Hospital of Shanxi Medical University, Shanxi Bethune Hospital, Shanxi Academy of Medical Sciences, Tongji Shanxi Hospital, Taiyuan, China

**Keywords:** Alzheimer’s disease, DNA methylation, CpG island, transcriptome, epigenetic age

## Abstract

**Objective:**

Alzheimer’s disease (AD) is a neurodegenerative disease characterized by neuropathology and cognitive decline and associated with age. The comprehensive deoxyribonucleic acid methylation (DNAm)-transcriptome profile association analysis conducted in this study aimed to establish whole-genome DNAm profiles and explore DNAm-related genes and their potential functions. More appropriate biomarkers were expected to be identified in terms of AD.

**Materials and methods:**

Illumina 450KGSE59685 dataset AD (*n* = 54) and HC (*n* = 21) and ribonucleic-acid-sequencing data GSE118553 dataset AD patients (*n* = 21) and HCs (*n* = 13) were obtained from the gene expression omnibus database before a comprehensive DNAm-transcriptome profile association analysis, and we performed functional enrichment analysis by Gene Ontology (GO) and Kyoto Encyclopedia of Genes and Genomes pathway enrichment analyses (KEGG). Three transgenic mice and three wild-type mice were used to validate the hub genes.

**Results:**

A total of 18,104 DNAm sites in healthy controls (*n* = 21) and AD patients (*n* = 54) were surveyed across three brain regions (superior temporal gyrus, entorhinal cortex, and dorsolateral prefrontal cortex). With the addition of the transcriptome analysis, eight hypomethylated-related highly expressed genes and 61 hypermethylated-related lowly expressed genes were identified. Based on 69 shared differentially methylated genes (DMGs), the function enrichment analysis indicated Guanosine triphosphate enzymes (GTPase) regulator activity, a synaptic vesicle cycle, and tight junction functioning. Following this, mice-based models of AD were constructed, and five hub DMGs were verified, which represented a powerful, disease-specific DNAm signature for AD.

**Conclusion:**

The results revealed that the cross-brain region DNAm was altered in those with AD. The alterations in DNAm affected the target gene expression and participated in the key biological processes of AD. The study provides a valuable epigenetic resource for identifying DNAm-based diagnostic biomarkers, developing effective drugs, and studying AD pathogenesis.

## 1. Introduction

Alzheimer’s disease (AD) is one of the most common age-related chronic neurodegenerative diseases that is characterized by progressive cognitive dysfunction and memory loss. The pathological features of AD involve the accumulation of amyloid-beta (Aβ) and neurofibrillary tangles. At present, there is no cure or treatment that can halt the progression of the disease. While the occurrence and development of AD is 70% dependent on genetic heterogeneity (Ballard et al., 2011), there are various risk factors, including cardiovascular risk factors (diabetes, hypertension, and obesity), age, and cognitive reserve (Daviglus et al., 2010; Mayeux and Stern, 2012). The involvement of specific epigenetic factors in the development of AD, including both pathological and physiological conditions, has become increasingly clear, (Berson et al., 2018; Perkovic et al., 2021) with deoxyribonucleic acid methylation (DNAm) being the most common epigenetic phenomenon. DNAm is crucial to regulating synaptic plasticity and maintaining the central nervous system (Perkovic et al., 2021). Previous research demonstrates that different regions of the brain exhibit distinct manifestations of AD (Wenk, 2003). Compared with the cerebellum, epigenetic perturbations in AD occur more frequently among multiple cortical regions, including the entorhinal cortex, prefrontal cortex, and temporal gyrus (Jager et al., 2014; Semick et al., 2019).

While previous studies successfully identify various DNAm differences associated with AD (Mastroeni et al., 2009; Chouliaras et al., 2013; Coppieters et al., 2014), existing research on methylation only identifies differentially methylated genes (DMGs) in a single brain region, while the relationship between DNAm and gene expression is not yet fully understood. Therefore, the present study aims to systematically assess multiple brain regions to identify DNAm alterations to drive the functionality of a transcriptome. A better understanding of the epigenetic mechanism of gene expression in AD could enable the discovery of more viable strategies for the prevention and treatment of the disease.

## 2. Materials and methods

### 2.1. Ribonucleic acid isolation, construction of nucleic acid library, and illumina sequencing

The feeding environment of SPF level mice should meet the following requirements: temperature 18∼22°C, Relative humidity: 40–70%, the humidity is 50∼60%. The experiment was carried out after 1 week of adaptive feeding. Mice were used three biological replicates, each of which comprised three frozen cortices from triple transgenic (3xTg) mice (females aged 5 months) that were processed for ribonucleic acid sequencing (RNA-seq). Meanwhile, the healthy control (HC) group contained three wild-type (WT) mice (females aged 5 months). The animals used in all experiments followed the National Institutes of Health guidelines, and all protocols were approved by the Institution Animal Care and Use Committee of Shanxi Medical University. The total RNA was extracted using TRIzol™ reagents (Invitrogen) in accordance with the manufacturer’s instructions, while the DNA was removed using DNase I reaction buffer (New England Biolabs). Prior to the RNA-seq, the quality of the RNA was assessed using the Agilent Bioanalyzer 2100 system, with 1.5 μg of the RNA sample subsequently taken for the sequencing procedure. The library preparations were sequenced on the Illumina system, with clean reads obtained by removing any reads containing adapter or ploy-N sequences and any low-quality reads containing > 50% bases with qualities of < 20 from the raw data. Meanwhile, reference genome and gene model annotation files were downloaded from the genome website directly, with paired-end clean reads aligned to the reference genome using Hisat2 v.2.0.5 software. FeatureCounts v.1.5.0-p3 was used to count the read numbers mapped to each gene.

### 2.2. Data collection

**FIGURE 1:**
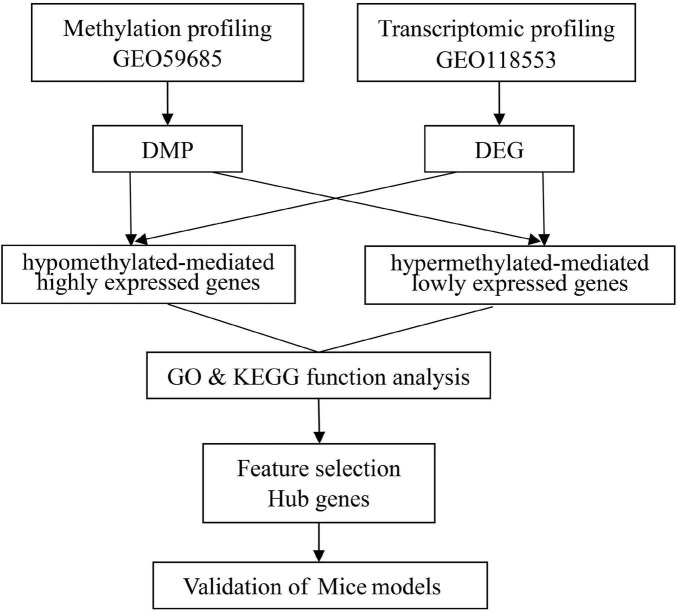
A flowchart of the study approach.

Illumina’s human methylation 450k array and specific gene expression profile datasets of AD were acquired from the gene expression omnibus database. In this search, the following three brain regions in a cohort of individuals were studied: the entorhinal cortex, the superior temporal gyrus, and the prefrontal cortex. The DNAm profiles of the AD (*n* = 54) and HC (*n* = 21) groups were obtained from the GSE59685 dataset (a cross-tissue analysis of methylomic variation in AD using samples from three independent human post-mortem brain cohorts), in addition to the transcriptome analysis data pertaining to AD patients (*n* = 21) and HCs (*n* = 13) from the GSE118553 individuals with intact cognition and neuropathology consistent with Alzheimer’s disease (AD) datasets. A flowchart of the study approach is shown in Figure 1.

### 2.3. Data pre-processing

To estimate the methylated intensity at individual 5’-cytosine–phosphate–guanine-3’ (CpG) sites, raw intensity files were preprocessed using Illumina’s (Taub et al., 2013) internal normalization probes and algorithms package, lumiMethyR, a pre-processing package that includes color balance adjustment, background level correction, and data normalization. The relative DNAm levels, represented as beta values (β), were derived from the intensities. Comparisons of AD-affected and normal brain tissues, differentially methylated probes (DMPs), differentially methylated regions (DMRs), and differentially methylated blocks (DMBs) were carried out using ChAMP (Tian et al., 2017). By applying the default setting, the raw data were filtered for probes with a detection value of non-CpG sites, the multi-hit probe list, or X and Y chromosomes. All DMPs with an adjusted *P*-value of < 0.05 were selected for further analysis.

The expression profile was preprocessed using the oligo package (Carvalho and Irizarry, 2010). Here, probes with more than one gene were removed, along with empty probes and duplicate gene symbols, with the maximum values regarded as the gene manifestation. The differentially expressed genes (DEGs) among the AD and HCs groups were screened using the limma R package (Federico and Monti, 2020). *P* < 0.05 set as the cut-off criterion for the DEGs.

### 2.4. Integration of transcriptome and deoxyribonucleic acid methylation

The hypermethylation of the DNA promoter region has traditionally been associated with transcriptional silence. Here, AD-related DMGs were identified, including hypomethylated-mediated highly expressed genes (hypo-HGs) and hypermethylated-mediated lowly expressed genes (hyper-LGs).

The finite element algorithm method (FEM) (Jiao et al., 2014) is a supervised clustering method that uses protein–protein interaction networks to perform a correlation analysis between Illumina Infinium 450k data and matched gene expression data to identify specific epigenetic gene modules or signaling pathways. Functional modules with methylation and gene expression levels undergoing significant changes can be determined using a FEM analysis.

### 2.5. Identification of cerebral-cortex-shared differentially methylated genes

To investigate common AD-related DMGs in the three cerebral cortex regions, Venn diagrams were created to reveal the degree of overlap between the DMGs in each brain region. A principal component analysis and unsupervised hierarchical clustering were used to examine the effects of the DMGs among the AD and HC groups. The Metascape database^1^ was used to conduct pathway and process enrichment analyses based on the DMGs, including gene ontology (GO) and Kyoto encyclopedia of genes and genomes (KEGG) biological functional enrichment analyses.

### 2.6. Identification of the hub genes

The Boruta algorithm was used as part of a feature selection method to identify the optimal diagnostic gene biomarkers for AD. The hub gene was evaluated in terms of the receiver operating characteristics (ROCs), with the diagnostic ability subsequently compared. Mice-based models of AD are widely used to study the pathogenesis of the disease and are critical for validating candidate biomarkers (Kursa and Rudnicki, 2010).

## 3. Results

### 3.1. Identification of differentially methylated 5’-cytosine–phosphate–guanine-3’ sites and differentially expressed genes in the three regions

**FIGURE 2:**
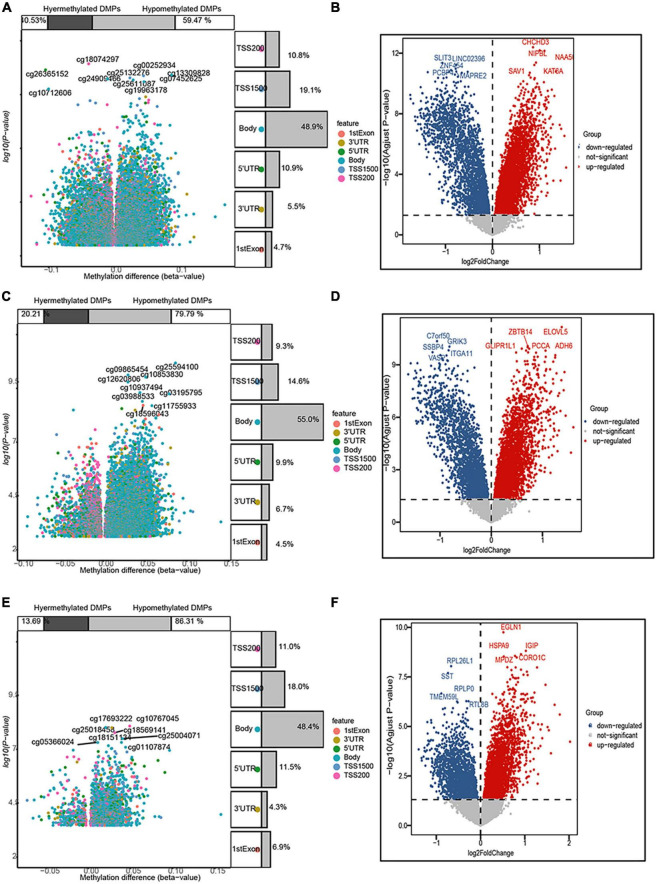
Volcano map of 5’-cytosine–phosphate–guanine-3’ (CpG) sites and differentially expressed genes (DEGs) after filtering. The three panels show the distribution characteristics of differentially methylated CpG sites and differentially expressed genes, respectively, in the **(A,B)** entorhinal cortex, **(C,D)** prefrontal cortex, and **(E,F)** superior temporal gyrus, as well as the ratio of hypermethylated to hypomethylated CpG sites.

In the entorhinal cortex, 26,350 DMPs were identified, which were annotated to 19,685 genes, including 7,978 (59.47%) with a higher methylation status, while 11,707 (40.53%) were hypomethylated. In the transcriptomic profiling dataset, 4,570 genes were up-regulated and 2,568 were down-regulated in the entorhinal cortex. In terms of the prefrontal cortex, 20,089 DMPs were identified and annotated to 15,835 genes, including 12,635 (79.79%) with higher methylation status, while 3,200 (20.21%) were hypomethylated, and 3,487 up-regulated DEGs and 2,382 down-regulated DEGs were identified. In terms of the superior temporal gyrus, 2,824 DMPs were identified, which corresponded to 2,082 genes, including 1,797 (86.31%) with a higher methylation status, while 285 (13.69%) were hypomethylated and 2,688 up-regulated DEGs and 1,929 down-regulated DEGs were identified. More CpG sites exhibited hypermethylation, while an analysis of the genomic locations of differentially methylated CpG sites revealed that place sites existed in the body for all brain regions, followed by TSS1500, 5’-UTR, and 1stExon (Figure 2).

### 3.2. Identification of Alzheimer’s-related cerebral-cortex-shared differentially methylated genes

**FIGURE 3:**
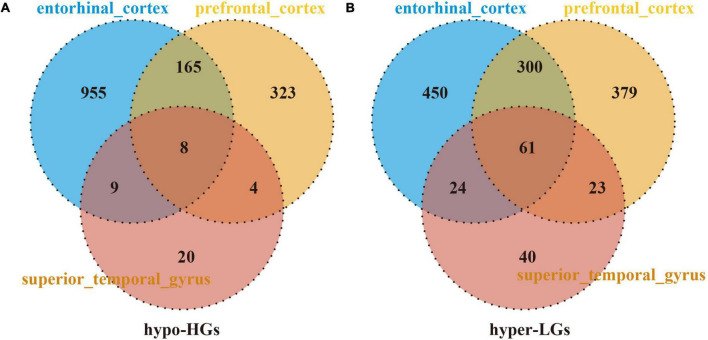
Identification of Alzheimer’s disease (AD)-related cerebral-cortex-shared differentially expressed genes (DMGs). The Venn diagram reveals eight hypomethylated-mediated highly expressed genes (hypo-HGs) **(A)** and 61 hypermethylated-mediated lowly expressed genes (hyper-LGs) **(B)**.

**FIGURE 4:**
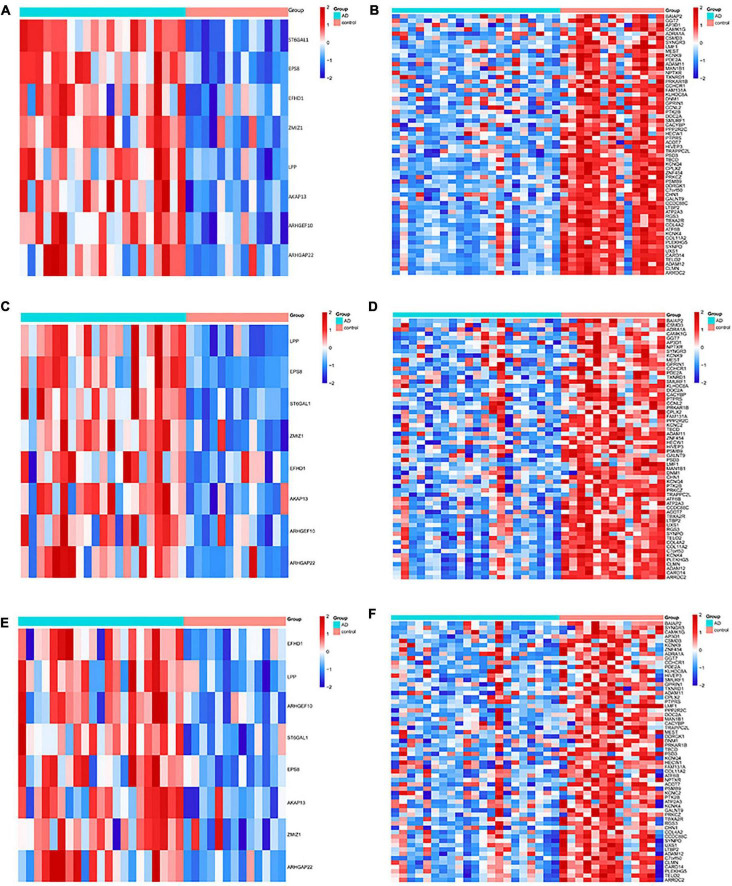
Heatmap of the differentially expressed genes (DMGs) among the Alzheimer’s disease (AD) and healthy control (HC) groups in three brain regions. The heatmap reveals eight hypo-HG and 61 hyper-LG expressions in the **(A,B)** entorhinal cortex, **(C,D)** prefrontal cortex, and **(E,F)** superior temporal gyrus. Each column represents a sample, and each row represents the transcription expression level of all the samples involved in one gene.

The DMGs were assessed by examining the DNAm and then any transcriptomic reversed alterations in the three brain regions. Using a Venn diagram, eight hypo-HGs and 61 hyper-LGs were revealed (Figure 3). Collectively, 69 aberrantly methylated DEGs were identified as AD-related cerebral cortex DMGs. An unsupervised hierarchical clustering heatmap of the shared DMG signatures revealed that the samples could be separated based on specific subject groups (Figure 4).

### 3.3. Functional enrichment of the differentially methylated genes

**FIGURE 5:**
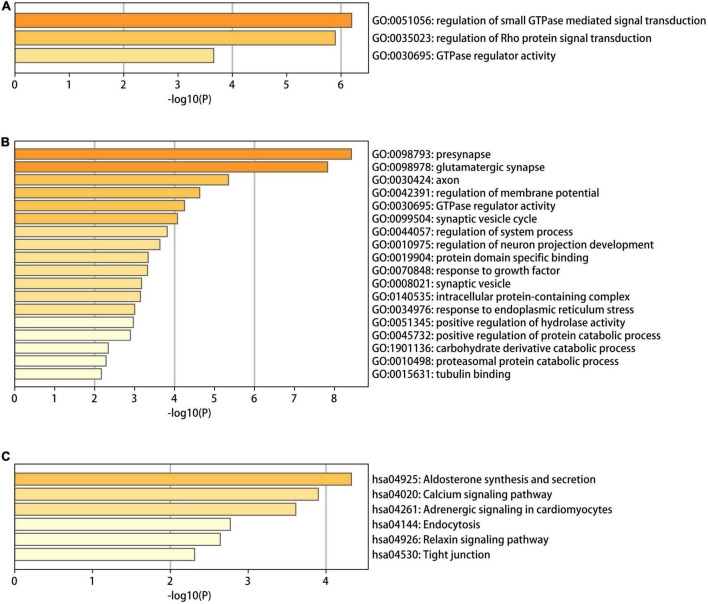
The gene ontology (GO) and Kyoto encyclopedia of genes and genomes (KEGG) functional enrichment analysis results for differentially methylated genes in Alzheimer’s disease (AD). **(A)** The GO terms in the eight hypo-LGs of AD; **(B,C)** the GO and KEGG terms in the 61 hyper-LGs of AD.

To identify the functional characteristics of the DMGs, an enrichment analysis was performed in terms of the shared DMGs (Figure 5). The GO analysis revealed that the hypo-HGs were enriched in Rho protein signal transduction, the regulation of small Guanosine triphosphate enzymes (GTPase) mediated signal transduction, and GTPase regulator activity, while the hyper-LGs significantly increased the GTPase regulator activity, the synaptic vesicle cycle, protein domain-specific binding, the positive regulation of the protein catabolic process, and the response to the growth factor. Meanwhile, the KEGG analysis results indicated that hyper-LGs were mainly involved in the relaxin signaling pathway, as well as endocytosis, tight junction functioning, and aldosterone synthesis and secretion. These outcomes indicated that methylation-driven DEGs may play essential roles in AD development.

### 3.4. Hub gene identification and validation of hub differentially expressed gene expression using the Alzheimer’s mice model

**FIGURE 6:**
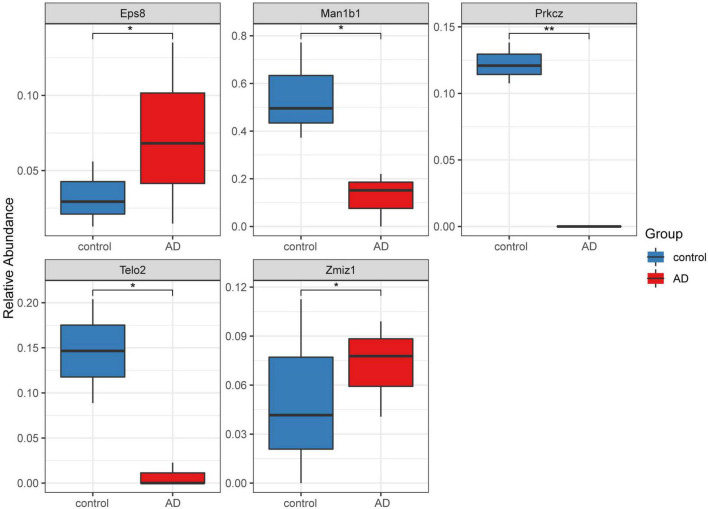
Five significantly expressed genes in the Alzheimer’s disease (AD) model mice compared with the control group. Five of the 14 genes had a significant expression level among the AD and control groups (*P < 0.05 and **P < 0.01). The red and blue boxes represent the AD and control mice, respectively.

A total of 16 DMGs were identified as optimal biomarkers for AD *via* feature selection using the Boruta algorithm. Following this, a ROC analysis was performed to evaluate the diagnostic utility of the 16 shared DMGs. Ultimately, a 14-gene combination was left behind, the area under the curve of which was ≥ 0.7, including three up-regulated and 11 down-regulated genes. Finally, in the AD mice model, We tested all the candidate genes and found that a total of five genes were confirmed with consistent differences as follows: epidermal growth factor receptor pathway substrate 8 (EPS8), zinc finger MIZ-domain containing 1 (ZMIZ1), telomere maintenance 2 gene (TELO2), protein kinase C zeta (PRKCZ), and mannosidase alpha class 1B member 1 (Man1b1) (Figure 6 and Table 1).

**TABLE 1:** The five genes, which have been validated by Alzheimer’s mice model, show high ability to discriminate patients with AD and healthy controls.

Gene	AUC (entorhinal _cortex)	AUC (frontal_cortex)	AUC (superior_ temporal_gyrus)
EPS8	0.917	0.965	0.97
ZMIZ1	0.853	0.886	0.886
TELO2	0.912	0.894	0.912
PRKCZ	0.864	0.886	0.864
MAN1B1	0.799	0.927	0.799

## 4. Discussion

The DNAm in various brain regions is an epigenetic factor that is interrupted in AD. Over the past few years, epigenetic therapies for AD have achieved a certain amount of success (Berson et al., 2018; Chatterjee et al., 2018). To further validate the relationship between DNAm and AD, a cross-brain region analysis was conducted. In the analysis, key differential genes among the AD and HC groups were identified by integrating the AD methylation data and gene expression measures in multiple brain regions.

The GO and KEGG analyses indicated that these DMGs were closely related to the small GTPase mediated signal transduction. Small guanosine triphosphatases (small GTPases) comprises five families: Ras, Rho, Rab, Arf, and Ran. The various roles of small GTPases super familys in AD pathogenesis have been extensively reviewed (Zhu et al., 2000; Bolognin et al., 2014; Arrazola Sastre et al., 2020, 2021), several studies have examined the relationship between Rho GTPases, amyloid precursor protein (APP) synthesis, and β-amyloid (Aβ) production in various cell lines (Boo et al., 2008; Wang et al., 2009). And Rho GTPases appear to contribute to the increase in β-amyloid (Aβ) production and resulting neurotoxicity (Aguilar et al., 2017). RhoA co-localized with hyperphosphorylated tau in neurofibrillary tangles (Huesa et al., 2010). Rho protein signal transduction pathway was also enriched in our results. Rab GTPases and Arf GTPases control the processing and trafficking of toxic peptides such as the APP, the axonal transport of proteins such as membrane receptors and autophagy. Therefore, pathological conditions lead to membrane trafficking defects (Kiral et al., 2018; Arrazola Sastre et al., 2020, 2021; Jordan et al., 2022).

The functional significance of the EPS8 in the brain has only just begun to be elucidated. The EPS8 activates the GTPase, Rac, leading to actin cytoskeletal remodeling (Offenhäuser et al., 2004). This is abundantly expressed in many brain regions, (Disanza et al., 2004) mainly in neurons in the cerebral cortex and hippocampus, which are two areas typically linked to higher cognitive functions (Offenhäuser et al., 2006). In addition, the EPS8 is enriched in the growth cone and filopodia of developing hippocampal neurons, where it down-regulates axonal filopodia formation (Menna et al., 2009). In the hippocampus, the conditional deletion of the EPS8 reduces the synaptic plasticity and impairs cognitive functioning (Wang et al., 2017). The synaptic vesicle cycle pathway in our study was directly enriched. In summary, the lack of EPS8 affects the synapse number but does not significantly impact the structural organization of the presynaptic compartment (Menna et al., 2013).

The ZMIZ1-related DNAm status is regarded as a molecular marker in multiple cancer types, including astrocytoma, bladder cancer, and renal cell carcinoma (Mathios et al., 2019). While the methylation differences among ZMIZ1 genes have not been well-studied in relation to AD, there is some circumstantial evidence for the association between AD risk and the expression of ZMIZ1. ZMIZ1 expression involves the regulation of neuronal and glial cell development through the Notch signaling pathway (Angulo-Rojo et al., 2013). In addition, the possible neurologic developmental function of ZMIZ1 may serve as a transcriptional coregulator in chromatin remodeling complexes (Carapito et al., 2019).

The TELO2 gene is essential to preserving genomic stability, and any disorder of the telomere maintenance mechanism results in telomere attrition (Kass-Eisler and Greider, 2000). Telomerase persists in the neurons of the adult human brain, where it may have a protective role against tau pathology (Boccardi et al., 2015). Forero et al. (2016) reported the presence of shorter telomeres in AD patients and highlighted the importance of the analysis of epigenomic markers associated with the risk of AD. Elsewhere, a multidomain lifestyle intervention facilitated telomere length maintenance among the subgroups of older people at risk of dementia and was linked to more pronounced cognitive intervention benefits (Sindi et al., 2021).

The internal promoter in the PRKCZ gene transcribes PKMζ, which is a constitutively active atypical kinase (Hernandez et al., 2003). Lee et al. (2013) targeted the exon 9 of the PRKCZ gene for developing experimental PRKZC^–/–^ in mice and observed that an absence of PKMζ does not impair the learning and memory in mice. However, several studies reported that PKMζ plays a significant role in maintaining synaptic long-term potentiation and long-term memory (Serrano et al., 2008). Therefore, the role of PKMζ in maintaining memory remains debatable and requires further discussion.

There are few reports related to the Man1b1 gene in AD. However, it has been widely reported in relation to Parkinson’s disease (Balducci et al., 2007; Tasegian et al., 2017; Federico and Monti, 2020; Zhang et al., 2021), with the activities of alpha (α)-mannosidase found to be substantially decreased in the cerebrospinal fluid of Parkinson’s disease patients (Balducci et al., 2007). However, Tasegian et al. (2017) recently reported that the α-mannosidase activity in cerebrospinal fluid mirrors the pathological changes in neurodegenerative disorders. Therefore, the α-mannosidase activity in AD is worthy of further exploration.

Finally, we tested all the candidate genes in the AD mice model to verify the biomarkeEPS8, ZMIZ1, TELO2, PRKCZ, and Man1b1, although the number of these genes is significantly less than that of candidate genes (possibly related to species differences), this has partly suggested that the genes we screened may be universal in the biological world, so these five genes can be well-verified. As for the accurate biological mechanism, it may need to be verified by subsequent experiments.

In conclusion, a combined analysis involving transcriptome and methylation was conducted. Further study of these hub genes in relation to AD and the pathways they participate in may provide new ideas for AD treatment.

This study had several limitations. First, only the DEGs have between verified in the two groups, while the methylation level of these genes was not verified. Therefore, it is necessary to further verify gene expression *via* a polymerase chain reaction or immunohistochemistry analysis.

## Data availability statement

The original contributions presented in this study are included in the article/Supplementary material, further inquiries can be directed to the corresponding author.

## Ethics statement

Ethical review and approval was not required for the study on human participants in accordance with the local legislation and institutional requirements. Written informed consent from the patients/participants or patients/participants’ legal guardian/next of kin was not required to participate in this study in accordance with the national legislation and the institutional requirements.

## Author contributions

GR, SS, S-XZ, and X-YL: conception and design of the research. GR, SS, YLv, Y-HW, and S-XZ: acquisition of data. GR, SS, and RZ: analysis and interpretation of the data. SS, Y-HW, and RZ: statistical analysis. GR, SS, and YLiu: writing of the manuscript. YLv, Y-HW, S-XZ, and X-YL: critical revision of the manuscript for intellectual content. All authors read and approved the final draft.

## Footnotes

1
www.metascape.org
