# Supplementary material for: Brain region-specific genome-wide deoxyribonucleic acid methylation analysis in patients with Alzheimer’s disease
Source: Front Mol Neurosci. 2023 Apr 13;16:971565. doi: 10.3389/fnmol.2023.971565 (PMC10133508; doi:10.3389/fnmol.2023.971565)
Supplement: Supplementary file 1 [file Data_Sheet_1.docx]

**Supplementary Materials**

**Epigenetic age acceleration and Alzheimer’s disease**

Recently, a highly accurate molecular biomarker of aging and the “epigenetic clock” was developed based on DNAm levels.^[1]^ Since AD is an age-related neurodegenerative disease, exploring the mechanisms behind the epigenetic clock can reveal its relationship with age-related cellular processes, such as senescence, epigenetic silencing, loss of mitochondrial function, and mutation accumulation.^[2, 3]^ By extension, the epigenetic clock can be used to examine how differences in DNAm age relate to AD. Therefore, the differences in AD epigenetic age among the AD patients and HCs were assessed, and the correlation between epigenetic and chronological age according to the brain region was examined using R software.

We aimed to functionally validate differences in DNAm associated with AD and, therefore, tested for AD case-control gene expression differences. We generated transcriptome data for mostly the same samples used in the DNAm analysis from the same brain regions. Then, we investigated the expression of genes near differentially methylated sites. Overall, DNAm was inversely correlated with gene expression. Data were obtained from (A) entorhinal cortex, (B) frontal cortex, and (C) superior temporal gyrus.

We analyzed the epigenetic age of AD patients and controls in three brain regions. The results showed that AD patients and controls showed a slowdown in epigenetic age, with no significant difference. DNAm age was highly correlated with the actual age at death, but the association between the age of DNAm and the exact age at death in the AD group was worse than in the normal control group. Therefore, we did not find the difference between the age of AD dementia and the age of DNAm, consistent with Morgan E. Levine et al.^4^. In addition, studies believed that epigenetic age has nothing to do with AD occurrence but only with the cognitive decline of people who are clinically diagnosed with AD^5^.

[1] Horvath S. DNA methylation age of human tissues and cell types [published correction appears in Genome Biol. 2015;16:96]. Genome Biol. 2013;14(10):R115. doi:10.1186/gb-2013-14-10-r115

[2] Klutstein M, Nejman D, Greenfield R, Cedar H. DNA Methylation in Cancer and Aging. Cancer Res. 2016;76(12):3446-3450. doi:10.1158/0008-5472.CAN-15-3278

[3] Zheng SC, Widschwendter M, Teschendorff AE. Epigenetic drift, epigenetic clocks and cancer risk. Epigenomics 2016;8(5):705-19. doi: 10.2217/epi-2015-0017 [published Online First: 2016/04/23]

[4] Salameh Y, Bejaoui Y, El-Hajj N. DNA Methylation Biomarkers in Aging and Age-Related Diseases. *Front Genet.* 2020;11:171.

[5] Levine ME, Lu AT, Bennett DA, Horvath S. Epigenetic age of the pre-frontal cortex is associated with neuritic plaques, amyloid load, and Alzheimer's disease-related cognitive functioning. *Aging (Albany, NY).* Dec 2015;7(12):1198-1211.

**Supplementary Figure 1: *epigenetic age of AD patients and controls in three brain regions.***

**Supplementary Figure 2: *Linear correlation Analysis between DNAm age and actual age. (A,B) entorhinal cortex, (C,D) prefrontal cortex, and (E,F) superior temporal***

***gyrus.***

**Supplementary Figure 3: *FEM analysis determined functional modules with methylation and gene expression levels undergoing significant changes. Each dot represents a gene in the module, with the color at the center of the dot representing the changing trend of differential methylation, and the color at the edge of the dot representing the changing trend of differential expression. (A)cerebellum, (B) entorhinal cortex, (C) prefrontal cortex, and (D) superior temporal gyrus.***
