# Supplementary figures and images for: Brain region-specific genome-wide deoxyribonucleic acid methylation analysis in patients with Alzheimer’s disease
Source: Front Mol Neurosci. 2023 Apr 13;16:971565. doi: 10.3389/fnmol.2023.971565 (PMC10133508; doi:10.3389/fnmol.2023.971565)

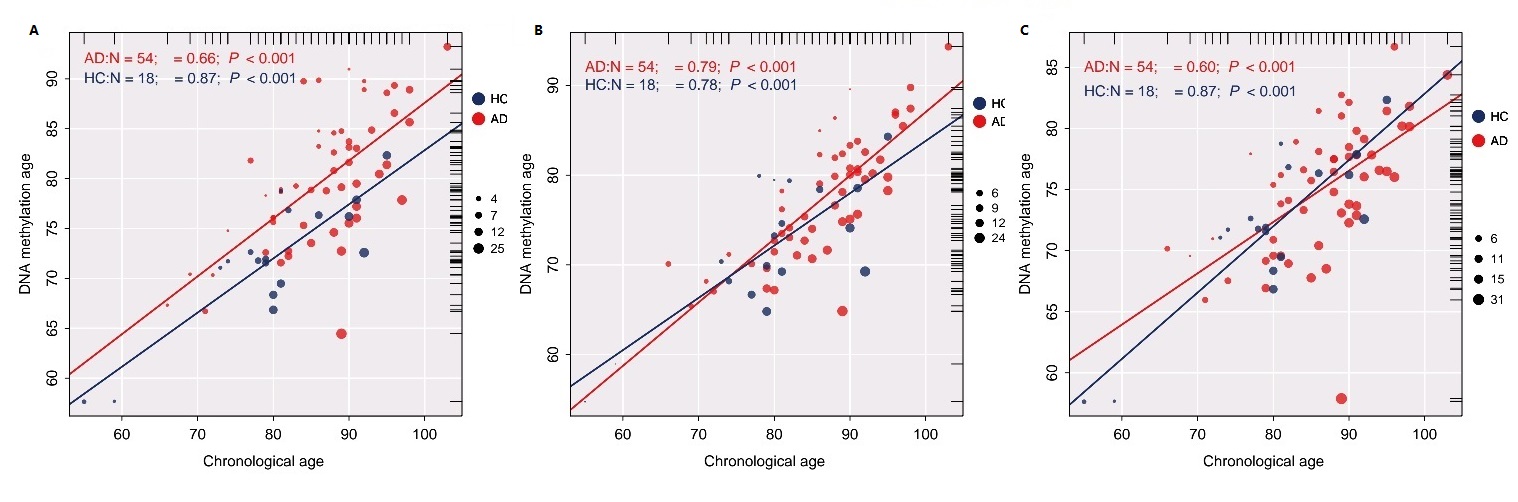

Supplement: Supplementary file 2 [file Image_1.JPEG]

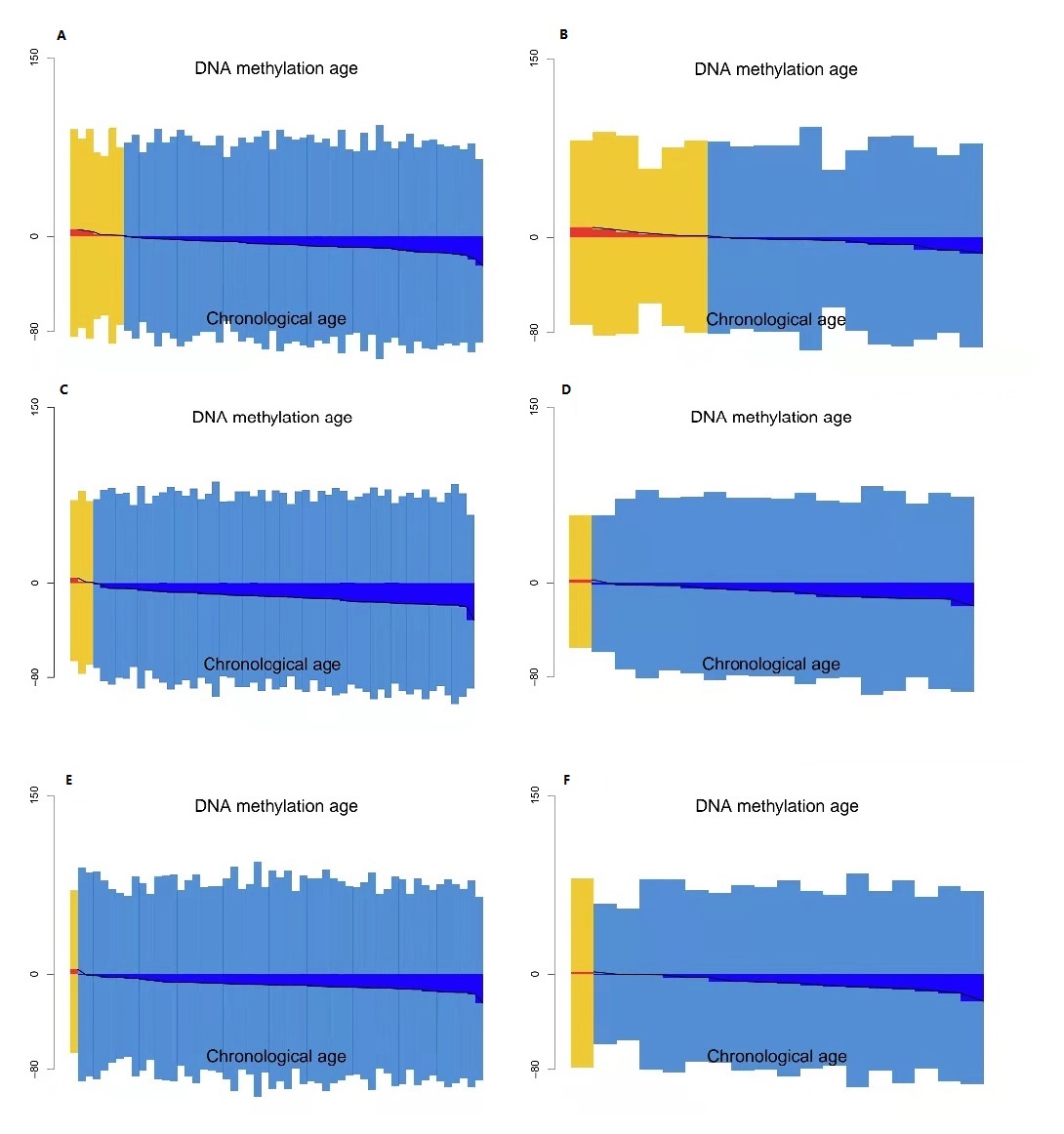

Supplement: Supplementary file 3 [file Image_2.JPEG]

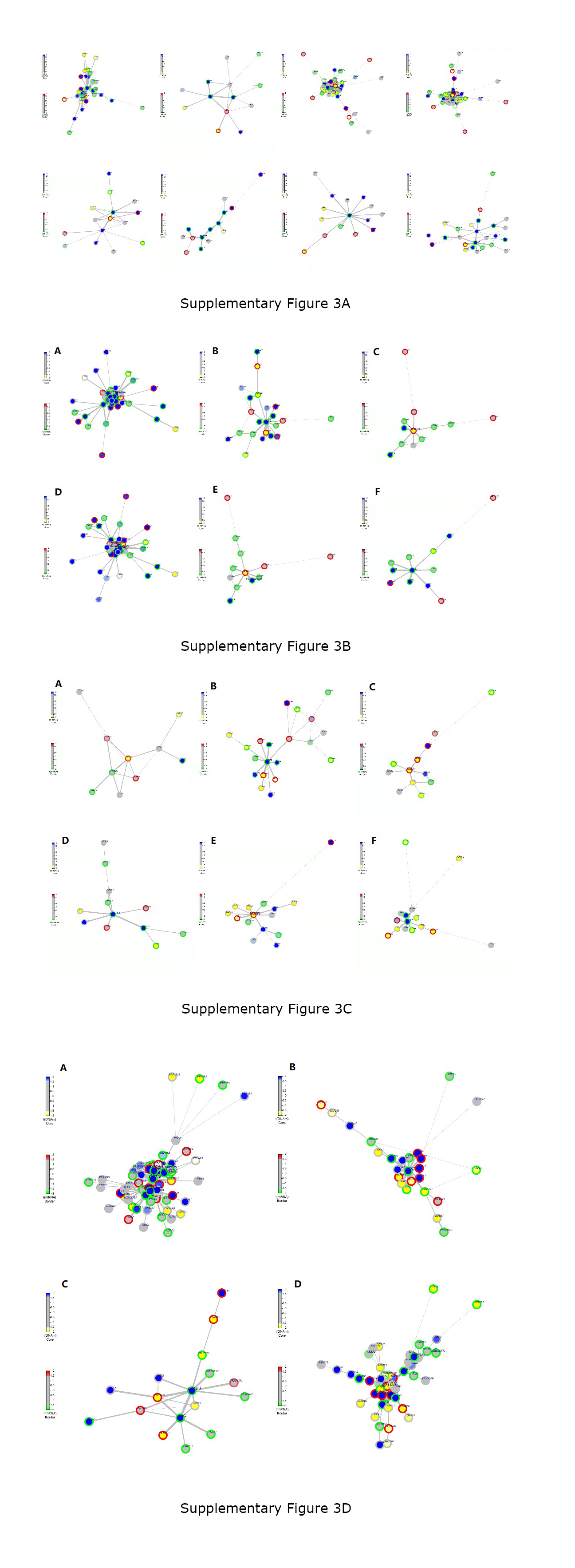

Supplement: Supplementary file 4 [file Image_3.JPEG]
